# Effect of Diet on Growth Performance of First Crab Stage *Callinectes sapidus* Rathbun, 1896 (Brachyura: Portunidae): A Comparison of Three Different Regimens

**DOI:** 10.3390/ani13071242

**Published:** 2023-04-03

**Authors:** Övgü Gencer, Hector Aguilar Vitorino

**Affiliations:** 1 Aquaculture Department, Ege University Faculty of Fisheries, 35040 Izmir, Turkey; 2 Institute of Marine and Environmental Technology, Columbus Center, University of Maryland Center for Environmental Science, 701 E. Pratt Street, Baltimore, MD 21202, USA; 3 Centro de Investigación en Biodiversidad para la Salud, Universidad Privada Norbert Wiener, Lima 15046, Peru

**Keywords:** *Callinectes sapidus*, growth, feeding, *Penaeus vannamei*, *Mastigoteuthis flammea*, *Oreochromis niloticus*

## Abstract

**Simple Summary:**

This study aimed to supply three foods to the crab *Callinectes sapidus* in its juvenile stage and compare their effects on its growth. For that, crab larvae were cultured from oviparous adult female crabs. The larvae (z1–z8) were fed with rotifers, previously cultured with microalgae and megalopae (Meg.) with live *Artemia salina* larvae, obtained from fresh cysts until they reached juvenile development (c1, first crab). Then, 270 animals (c_1_) were analyzed in three groups of 90, with different diets: shrimp (*Penaeus vannamei*; Group 1), squid (*Mastigoteuthis flammea*; Group 2), and tilapia fish (*Oreochromis niloticus*; Group 3). After 90 days of feeding regimens, the sizes of juvenile crabs were measured by microscopy, and the following relationship was found (*p* < 0.001): Group 1 (20.8 ± 0.7) > Group 2 (14.5 ± 0.9) > Group 3 (10.4 ± 0.6).

**Abstract:**

This study aimed to supply three foods to the crab *Callinectes sapidus* in its juvenile stage and compare their effects on its growth. For that, crab larvae were cultured from oviparous adult female crabs. The larvae (z_1_–z_8_) were fed with rotifers, previously cultured with microalgae and megalopae (Meg.) with live *Artemia salina* larvae, obtained from fresh cysts until they reached juvenile development (c_1_, first crab). Then, 270 animals (c_1_) were analyzed in three groups of 90, with different diets: shrimp (*Penaeus vannamei*; Group 1), squid (*Mastigoteuthis flammea*; Group 2), and tilapia fish (*Oreochromis niloticus;* Group 3). After 90 days of feeding regimens, the sizes of juvenile crabs were measured by microscopy, and the following relationship was found (*p* < 0.001): Group 1 (20.8 ± 0.7) > Group 2 (14.5 ± 0.9) > Group 3 (10.4 ± 0.6). The nutritional factor played an essential role in this size differentiation. This intelligent and differentiated feeding strategy showed us that shrimp could be an essential source for the growth of crabs in the juvenile stage. This new approach to safe and efficient roost feeding can classify crabs by size for further hormonal, molting, and reproductive studies.

## 1. Introduction

The blue crab *Callinectes sapidus* Rathbun, 1896 (Arthropoda: Brachyura: Portunidae), is a sizeable epibenthic omnivore decapod crustacean species native to the Western Atlantic region and was introduced accidentally or intentionally into both Asia and Europe [1]. The blue crab represents the most valuable fishery in the Chesapeake Bay, which is considered an iconic species and a crucial component of the ecosystem. In their juvenile stage, they are easy prey for other vital predators such as striped bass (*Morone saxatilis*) [2], red drum (*Sciaenops ocellatus*), Atlantic croaker (*Micropogonias undulatus*) [3], and adult blue crabs [4]. The adult blue crab is a generalist predator and scavenger, feeding on a wide variety of benthic invertebrates, such as polychaetes, clams, crustaceans, some vertebrates as small fish, and plant material. This species supports one of the region’s most lucrative commercial and recreational fisheries. Therefore, it is of ecological and economic importance to maintain a stable population of blue crabs to preserve the sustainability of the resource [5,6].

Being part of the leading fishing economy of the Chesapeake Bay, the Mid-Atlantic states of Maryland, Virginia, and North Carolina, blue crab production has been steadily declining. According to the Maryland Department of Natural Resources, the population has decreased in the last ten years by 75% (227 million, 2022) [7]. Although more alarming may be the drop in harvests of breeders in the Chesapeake Bay (81%) [7,8]. Similarly, in North Carolina, adult crabs and postlarval stages decreased by 74% and 71%, respectively, in the last 20 years [9,10]. The fishing factor and destruction of habitats where the blue crab breeds have led the populations of these animals to a crisis.

In that sense, strategies to reverse this declining trend of blue crabs must be taken, including regulating fisheries to protect the producing populations, spawning, and abundance of larvae [8,11]. Therefore, blue crab is a great candidate for population enhancement, which led us to try various approaches to replenishing its severely depleted population [9,12,13,14]. Although blue crab aquaculture systems were developed [15], being a more complex process than other crabs that only go through five (mud crab *Scylla serrata*) [16] or four (blue swimming crab *Portunus pelagicus*) [17] zoeal larval stages, the blue crab has up to eight significantly shorter zoeal stages [18]. Furthermore, multiple life stages require strict feeding protocols for their more elaborate larvae, mainly in the final stage of zoeae, and megalopa, as they become highly cannibalistic [5], and it is even necessary to implement isolated hatchery systems.

Inseminated, mature female blue crabs migrate to the Chesapeake Bay to higher salinity waters at the mouth, producing a dense orange mass of eggs (a sponge: millions of eggs). Eggs remain closely attached to the female’s abdomen for approximately two weeks. The initially orange sponge becomes progressively darker as the growing embryos develop eyes. Upon hatching, larval crabs (zoeae) look more like shrimp. They are planktonic and employ currents to move back in and up the Chesapeake Bay. After eight zoeal stages (z_1_–z_8_), larvae morph into lobster-like megalopae (Meg). Settlement begins at this. The tiny first crab (c) stage (from tip to tip of the carapace spikes: 1–2 mm) comes after the megalopae stage. After that, they travel a long distance up the Bay and into marshy tributaries, where they will spend most of their lives. Finally, juvenile metamorph to adult passing in approximately 18–21 stepwise increments of molts [5,19].

Knowing the diet of a species is essential to understand its nutritional requirements and its interaction with other animals [20]. This information is also beneficial for implementing successful rearing systems. Crabs can inhabit many habitats in a wide variety of geographic areas, which is reflected in the type of foods they eat [21]. Crabs are opportunistic omnivores with preferences for foods of animal origin and a solid predatory tendency [22]. A wide variety of species follows this feeding pattern; their specialization in a single type of food is not shared. Crabs can deal with various foods, but they are primarily carnivorous [21]. Another essential phenomenon to know about crustaceans is cannibalism [15]. Regardless of the size of the species, predation by fitter or larger animals on smaller or more vulnerable ones is a factor influencing population dynamics [23]. Tiny habitat that prevents dispersal, high population density, scarcity of food, or structural simplicity of the habitat can encourage cannibalistic behavior, mainly in juvenile crabs, both in natural and cultured environments, affecting population dynamics. In addition, it can alter the distribution of individuals [14,15]. These effects have already been reported in species such as *Cancer magister* [24], *Callinectes sapidus* [15], *Chionoecetes opilio* and *Carcinus maenas* [25]. Therefore, implementing individual crab hatchery systems would be an alternative to culture crabs.

This study aimed to examine the effect of three different feeding regimens using *Callinectes sapidus* crabs in their juvenile stage. Larval (z_1_–z_8_) and postlarval (Meg) feeding with a combination of rotifers (fed microalgae) and *A. salina* larvae were taken into account to obtain c_1_-stage animals with good phototactic activity [15,26]. In addition, independent culture systems were implemented for this study in order to avoid cannibalism.

## 2. Materials and Methods

### 2.1. Animal Collection

Primiparous adult female *C. sapidus* were collected in the Chesapeake Bay during the fall seasons of 2013 to 2015 and reared in the blue crab hatchery in the Aquaculture Research Center (ARC), Institute of Marine and Environmental Technology (IMET), Baltimore, MD, USA. Animals were kept in captivity in five closed circulation aquaculture system tanks (90 cm W × 110 cm H × 60 cm D) at 8 h light and 16 h dark photoperiod conditions. These tanks contained 300 L of artificial seawater (ASW) with 30 ppt at 22 ± 1 °C.

ZooQuatic Lab (Baltimore, MD, USA) monitored the water quality daily. For the feeding study, eggs were obtained from pregnant adult females with carapace widths of 130 ± 4 mm (*n* = 10) [27,28]. The animals were fed daily with 5–10 g (or 10% of their body weight) of a piece of frozen squid.

Female crabs carrying imminently hatching embryos, previously analyzed [29], were transferred into 50 L ASW, 30 ppt broodstock tanks. All the animals obtained were produced from November 2018 to September 2019. To obtain the larvae, it was necessary to manipulate the blue crab females by increasing the temperature (22 ± 0.5 °C) and photoperiod (~14 h of light) to promote ovulation [9]. This environmental regimen was carried out for 4–5 months. This regimen was performed in a closed system with continuous water recirculation to keep blue crab females healthy for larval release. The crabs were kept in containers filled with sand underwater to facilitate feeding and cleaning of the tank and general health conditions. During their feeding (8–9 am each day) [15,29], it was necessary to conduct it in the presence of red light to reduce the degree of stress caused by the white light of the external environment [9,15,30].

### 2.2. Rearing of Larval and First Crabs

Immediately after hatching, the zoeae (stage z_1_) with the highest phototaxis activity were collected: animals that swam toward a high-intensity light (110 lumens). The zoaeas were kept in a 500 mL glass beaker with ASW, 30 ppt with aeration, at 22 ± 1 °C for 2 h until before collection. Then, to avoid cannibalism during experiments, the zoaeas were placed in 12-well flat plates (one animal per well) with 3 mL of ASW (30 ppt) previously filtered with 0.22 µm membranes (Millipore, Bedford, MA, USA). The animals were fed daily with rotifers (50 u per well) between 9:00 and 10:00 h until megalopae (Meg) development under a 12:12 light: dark photoperiod. Previously, the rotifers were cultured and fed with 2.5 mg (dry wt.) L^−1^ of marine-microalga *Nannochloropsis* sp. (Reed Mariculture, CA, USA) [15]. The water was exchanged daily, carefully transferring (using a plastic micropipette, 1.5 mL) the zoaeas to a new plate for approximately 20 days (stage z_8_) or until all animals transformed into megalopae (stage Meg) [29,31].

The animals in the megalopae stage were fed under a regimen of live *Artemia salina* larvae (20 individuals per day) [15]. Artesmia larvae were obtained from the hatching of *A. salina* cysts (Artemia international, Fairview, TX, USA) cultured for 20–22 h at 25 ± 1 °C, in ASW 30 ppt, under a continuous aeration system and with lighting (110 lumens) [30]. Artemia culture was performed daily. However, they were not fed during the entire process, and the larvae were kept and transported in glass containers and ventilated before each use.

Once the first crab (c stage) was obtained, after 10 days, from the Meg stage, the animals were fed under regimens of 0.001 g per mL: shrimp *Penaeus vannamei* (Group 1), squid *Mastigoteuthis flammea* (Group 2), or tilapia *Oreochromis niloticus* (Group 3). Each group corresponded to 90 animals, and each of the 12-well plates contained 10 *Callinectes sapidus* crabs.

### 2.3. Growth Experiment

#### 2.3.1. Raw Material

The animal food (shrimp, squid, and fish) was purchased frozen (−20 °C) and packed in polyethylene bags, which were supplied by aquarium co-op, MD, USA (Supplementary Materials Table S1). It was used seven days after processing and, according to the supplier, is valid for six months. Small frozen portions of food were thawed for daily feeding.

#### 2.3.2. Feeding Regimen

Using the same water system, rearing, and feeding of the zoaeas (z_1_ → z_8_) and megalopae (Meg), stage c (first crab) crabs were reared in the same 12-well flat-bottom microplates. Once the first stage of crab (c) was obtained, the diet was modified (shredded): shrimp, *Penaeus vannamei* (Group 1); squid, *Mastigoteuthis flammea* (Group 2); and fish Tilapia, *Oreochromis niloticus* (Group 3).

Then, 270 first crabs *Callinectes sapidus* (c_1_ = 1.5 ± 0.2 mm, day = 0) were collected and divided into three groups of 90 (1, 2, and 3). The crabs were fed from the c_1_ stage or first crab. Groups A, B, and C received a diet of shredded shrimp (without exoskeleton), various sizes of frozen squid (only tentacles), and shredded fish (only muscle), respectively (Table 1). The rations were kept constant during the growth test days. Feeding was performed twice daily between 8:00 and 9:00 am and 2:00 and 4:00 pm for 90 days (day = 90). Then the crab was measured across the broadest part of the shell from tip to tip of the carapace spikes; the growth of the animals was followed by microscopy using a stereoscopic microscope (AmScope SE306R-PZ-P, Irvine, CA, USA). The experiment was carried out in 9 plates of 12-wells for each group, each containing 10 individuals of the first crab, carefully repeating each individual’s experimental conditions [15].

Crabs were fed twice a day with 3 mL of a suspension of 0.001g/mL of shredded diet. The food mixed with the water was added so as not to modify the final volume of water (3 mL). Each container (plate) was changed with a new one, and the water was changed before each feeding (twice a day), maintaining the water quality parameters (Table 2). The animals’ carapace measurements were made at the beginning of the experiment (first crab, day = 0) and the end of the experiment (juvenile crab, day = 90).

### 2.4. Statistical Analysis

The experiment had nine independent replicates performed at the same time, and the data were analyzed using SigmaStat 3.2 software (Systat Software Inc., San Jose, CA, USA) with one-way ANOVA followed by Tukey’s post hoc test, which was used to compare the growth of the animals under the three types of feeding. The level of significance adopted was 5%. The figure and data fitting were produced using OriginPro version 2019 (OriginLab Co., Northampton, MA, USA). Data are presented as means ± SD.

## 3. Results and Discussions

This work aimed to collect essential information on the differences in the growth of the first crab when different diets are administered. This stage (c_1_) of the crab *Callinectes sapidus* is one of the fundamental stages for developing the juvenile and adult crab. Our study was carried out in the IMET facilities (University of Maryland, Baltimore, USA), with all the necessary technology, and designed for the intensive production of crustaceans in captivity. The c_1_ stage of crab development is crucial since they are transferred to systems in growth tanks [15]. Therefore, the crabs must reach 15–20 mm to conduct subsequent repopulation studies. These animals were farmed and bred individually due to impending cannibalism, mainly in the molting stage [5]. Hence, this work seeks to provide information not yet reported in the literature regarding the type of food that can be beneficial for growth at this crab stage.

**Figure 1:**
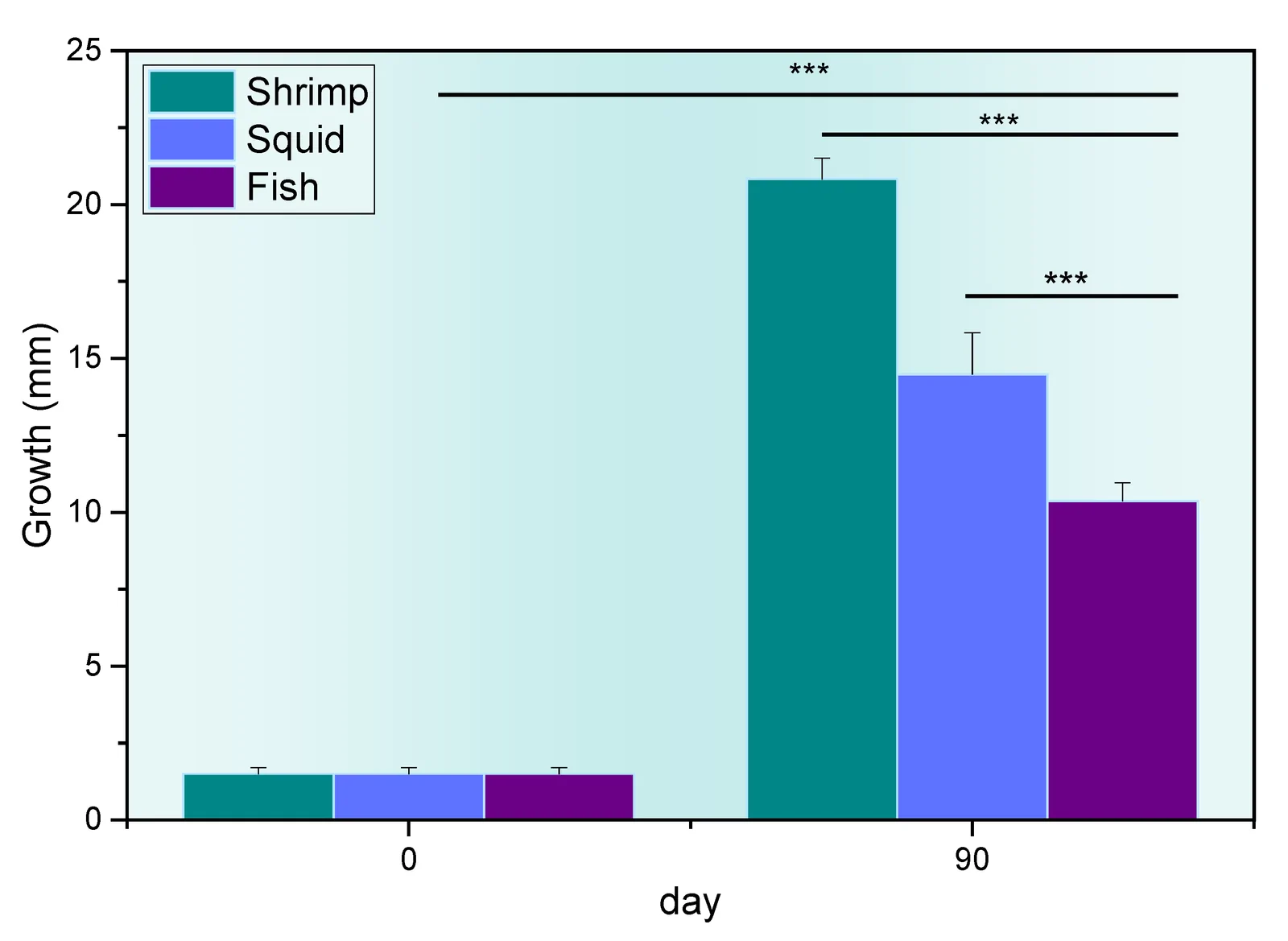
Growth rates of crabs according to different feeding regimes: shrimp *Penaeus vannamei* (Group 1), squid *Mastigoteuthis flammea* (Group 2), and tilapia *Oreochromis niloticus* (Group 3). Feeding period = 90 days. Feeding times = twice a day. Amount of food = 0.001 g per mL. *** *p* < 0.001.

This study was carried out with careful animal handling to avoid mortality in each of the wells (mortality = 0%), obtaining continuous growth data during the 90 days of growth (first crab → juvenile). During that time, it was observed that our crabs showed divergent growth according to the type of feeding (Table 1, Figure 1).

Inferior water quality in aquaculture systems is considered one of the most critical factors in captive crab breeding [32]. As a result, water quality parameters can affect crab growth rates and lead to a higher mortality rate. Maintaining and monitoring the physical (temperature) and chemical (salinity, dissolved oxygen, pH, ammonia, nitrite, etc.) variables were essential to maintain the excellent health of the animals. Our rearing systems always maintained an adequate quality of synthetic seawater (Table 2). The increase in the nitrogenous concentrations of compounds in the solution was diluted with fresh and seawater (<1 ppm). Furthermore, the presence of sediments, microorganisms (*Euplotes* sp., *Zoothamnium* sp. or *Vorticella* sp.), nematodes, copepods, or dinoflagellates was not observed in the synthetic water after treatment with 0.22 µm filters (Millipore^TM^).

The increase in temperature and the light photoperiod were necessary to promote ovulation in the crabs. The water recirculation system efficiently kept the hatching tank clean [9]. The sand played an essential role in simulating the crab’s habitat to facilitate the release of eggs. The red light helped reduce stress in the animals after exposure to the aquaculture room’s white light [9,15,30]. During the growth study, the animals were immersed in water all the time. Crabs were avoided in stress due to possible hypoxia from exposure to air [33], as this may interfere with their mortality. When removing the residues, no difference was observed between the food and the shells because the animal crushed them. The molting stages were difficult to follow when changing water and feeding twice daily.

Once the larvae (zoeae, z_1_) were obtained, they were kept separately in each well in flat 12-well plates to avoid cannibalism that can lead to up to 43% mortality in the Meg. stage [15,34]. Raising crabs in low densities can favor the survival rate; for example, for the species *P. camtschaticus*, the decrease in crab density increased the survival rate from 65% to 94% [35]. On the other hand, strict compliance with feeding with live animals (rotifers and *Artemia nauplii*) has excellent survival rates of up to 74% for *C. sapidus* [15] and 61% for *P. trituberculatus* [11,35,36]. Not only can 100% survival be achieved with well-tuned feeding, as this study shows, but different feeding regimens can achieve a clear difference in growth (Figure 1). It should be noted that the low levels of mortality in the larval (z_1_–z_8_), megalopa (Meg.), and juvenile (c) stages were due to the fact of them being reared in separate and differentiated spaces [15,19]. Additionally, cannibalism interferes with the total survival count since no dead animals were observed, but a drop in the number of megalopae was observed [34]. This observation reinforced our differentiated hatchery methodology to obtain animals of homogeneous sizes until obtaining the first crab (c).

The growth of animals fed in groups is difficult to control due to a largely heterogeneous development. That is, one animal can be fed with the ration of another, generating differences in the size of the population. In addition, animals at each stage may have different feed requirements over a long rearing period. When carrying out our study with animals in independent spaces, it was possible to eliminate this interference, thus observing differentiated growth according to the type of diet. Swimming space also played an important role, 1 animal/3 mL (100% survival); this aided in efficient molting and cleaning of the containers. The food type and the amount offered slightly affected crab behavior (swimming ability). Mainly for animals that consumed shrimp and squid. This behavior is due to the type of food or its nutritional level [23], which can favor molting and, therefore, growth [15].

The nutritional value differs mainly in the content of calcium, nitrogen, and phosphorus (shrimp > squid > fish) [37] Calcium content in the diet can increase the growth efficiency of crabs, as shown in this study (Group 1 > Group 2 > Group 3) (*** *p* < 0.001). Our results showed the same growth response as in the *Scylla serrata* [38], *Eriocheir sinensis*) [39], and superfamily *Grapsoidea* [40] species. Different diets with nutritional content rich in calcium can alter the frequency of molting and therefore promote the growth of crabs [41].

The crabs that obtained more significant growth showed a greater tendency to swim and stay in the upper part of the water column. This was due to their ability to swim, which is related to their development and growth to reach their food quickly [9,29]. The diet provided was always maintained by weight per volume unit and not by the animal’s size to keep the same amount of food for comparative purposes. Offering this differentiated diet allows us to simplify the complexity of the diet in the future. It should be noted that we did not focus on a molt study but on growth from our experimental approach.

Raising the crabs individually was critical to studying growth with different types of food. Although it can be tedious work, this rearing method can be beneficial for studying different growth rates without interference. Cannibalism, mainly in the initial stages of the crab, can be a significant interferer in this type of study [23]. Raising crabs in large populations with high predatory capacity can make growth studies complex regarding size distribution. Natural environments or habitats may be the best place for a crab growth study, but they may be exposed to microorganisms that can be detrimental to the health of each animal [23]. Carrying out this study in independent systems and with water recirculation systems to obtain the larvae was essential to achieve a minimum mortality rate. Using synthetic water free of harmful organisms for the animal was also necessary to avoid mortality in our crabs. At all times, we concentrated on observing the growth rate with each type of diet provided to the crab, postponing the information on the number of molts for later studies. On the other hand, the most convenient type of food can be observed to achieve better growth of the *Callinectes sapidus* crab from its post-larval stage.

## 4. Conclusions

This study shows for the first time that *Callinectes sapidus* crabs, reared and fed individually, can show differences in growth depending on the feeding from the first crab to the juvenile stage. This trial can be performed at any time of the year and with reasonable survival rates compared to other species in high-density aquaculture systems. The individual hatchery mode, avoiding cannibalism, made it possible to attain 100% survival in the larval stages, zoeas, megalopa, and first crab. The intelligent and differentiated feeding strategy showed us that shrimp is a food source that promotes growth in *C. sapidus crabs*. This new approach to safe and efficient refuge feeding can classify crabs by size for further hormonal, molting, and reproductive studies.

## Figures and Tables

**Table 1:** Growth of crabs (3 groups × 90 animals) for 90 days with shrimp *Penaeus vannamei* (Group 1), squid *Mastigoteuthis flammea* (Group 2), and tilapia *Oreochromis niloticus* (Group 3). (Average ± SD, *n* = 3 for three independent experiments).

*n* = 270 Animals	Group 1 (*n* = 90)	Group 2 (*n* = 90)	Group 3 (*n* = 90)
Diet (0.001 g/mL)	Shrimp	Squid	Fish
Size (day = 0), mm	1.50 ± 0.20		
Size (day = 90), mm			
Plate 1 *	21.0 ± 1.50	11.9 ± 1.20	11.1 ± 0.40
Plate 2	20.4 ± 1.00	16.1 ± 1.10	10.5 ± 1.40
Plate 3	21.2 ± 1.60	12.4 ± 1.10	9.90 ± 1.60
Plate 4	21.2 ± 0.90	14.5 ± 0.40	11.1 ± 0.40
Plate 5	22.1 ± 1.20	15.5 ± 2.20	10.6 ± 0.90
Plate 6	20.0 ± 2.10	15.3 ± 0.70	9.50 ± 1.40
Plate 7	20.1 ± 1.00	14.4 ± 1.30	11.0 ± 2.20
Plate 8	21.4 ± 1.30	15.2 ± 2.10	9.70 ± 1.20
Plate 9	20.1 ± 1.40	15.1 ± 1.80	9.90 ± 1.30

* Each 12-well plate contains 10 *Callinectes sapidus* crabs in 3 mL of synthetic seawater.

**Table 2:** Optimal aquaculture water quality parameters for the growth experiment.

Water Quality/Physicochemical Variables	Optimal Level
Ammonia (NH_3_, NH_4_^+^) and nitrite (NO_2_^−^)	<1 ppm
Dissolved oxygen (DO)	90% saturation (approx. 6.7 ppm)
pH	7.7–8.0
Salinity	30 ppt
Temperature	22 ± 1 °C

## Data Availability

All data are included in the manuscript and Supplementary Materials, and further details are available upon request.

## Supplementary Materials

The following supporting information can be downloaded at: https://www.mdpi.com/article/10.3390/ani13071242/s1, Table S1. Nutritional content: shrimp, squid, and fish.
