# Supplementary material for: Effect of Diet on Growth Performance of First Crab Stage Callinectes sapidus Rathbun, 1896 (Brachyura: Portunidae): A Comparison of Three Different Regimens
Source: Animals (Basel). 2023 Apr 3;13(7):1242. doi: 10.3390/ani13071242 (PMC10093128; doi:10.3390/ani13071242)
Supplement: Supplementary file 1 [file animals-13-01242-s001.zip › animals-2075198-supplementary.pdf]

animals

Effect of Diet on Growth Performance of First Crab Stage *Callinectes Sapidus* Rathbun, 1896 (Brachyura: Portunidae): A Comparison of Three Different Regimens

Övgü Gencer[1,2,*] and Hector Aguilar Vitorino[3,*]

Table S1. Nutritional content: shrimp, squid, and fish.

	Nutritional content		
	Shrimp (85 g), 60 Cal	Squid (85g), 80 Cal	Fish (85 g), 110 Cal
Total Fat	1.0 g	1.0 g	2.5 g
Cholesterol	105 mg	200 mg	50 mg
Sodium	480 mg	35 mg	50 mg
Carbohydrate	1.0 g	3.0 g	0 g
Protein	12 g	13 g	22 g
Calcium	52 mg	46 mg	13 mg
Iron	0 mg	0.72 mg	0.95 mg
Potassium	96 mg	0 mg	396 mg

*The shrimp is free of its exoskeleton, the squid only includes tentacles, and the fish only includes muscle.
